# Linear Low-Dose Extrapolation for Noncancer Responses Is Not Generally Appropriate

**DOI:** 10.1289/ehp.0800329

**Published:** 2009-04

**Authors:** Lorenz R. Rhomberg

**Affiliations:** Gradient Corporation, Cambridge Massachusetts, E-mail: lrhomberg@gradientcorp.com

I disagree with the assertion of White et al. (2009) that linear low-dose extrapolation is generally appropriate as a default for noncancer, as well as cancer, end points. Such a stance would radically alter a basic tenet of toxicology and risk analysis, and any such course should be considered only after rigorous and broadly based examination of the scientific principles involved.

White et al. (2009) offered only a cursory summary of arguments that were discussed in a 2007 workshop, but any fuller discussion that may have occurred in that workshop was not recounted. Continual revisiting of the scientific thinking under lying risk assessment policies is valuable, but a change of this nature—which would depart from decades of well-established practice—needs to be carefully and critically examined.

I believe that most observers would find fault with all of the proffered lines of reasoning that White et al. (2009) cited in advocating that noncancer dose–response relationships should be treated as linear. Although harmonization of cancer and non cancer toxicity assessment holds some value (regarding commonality of pharmacokinetics and, potentially, elements of modes of action), there are still fundamental differences between carcinogenicity on the one hand, in which the probability of constellations of rare events (that get rarer with lower doses) drives the dose–response function, and most noncancer responses on the other, in which the dose–response function hinges on the degree of perturbation of physiologic and homeo static processes (which becomes less pronounced and less efficacious with lower doses) (Rhomberg 2004).

The argument of White et al. (2009) that epidemiologic studies often show no thresholds, even for end points having thresholds in animal studies, is readily attributable to the small range of exposure levels and the approximate nature of exposure measurements in most human studies; these artifically flatten apparent dose–response curves and tend to make any dose-related effect (even those that are truly threshold in nature) look more or less linear as an artifact of the analysis.

Heterogeneity in sensitivity and in modifying factors among people in the target population may tend to broaden the dose–response relationship, but it does not linearize it, as White et al. (2009) asserted; indeed, the logic they invoked (the combined effect of variation in many modifying factors) leads to the expectation of a cumulative log-normal dose–response function, which is always nonlinear, rather than a linear one.

Similarly, when examined rigorously, the invocation by White et al. (2009) of the principle of additivity to background fails to support general linearity; unless it is framed in the discussion of a specific mode of action, the additivity-to-background argument amounts to begging the question—assuming the hypothetical existence of an underlying and rate-limiting no-threshold mechanistic effect to argue for linearity of the end result caused by that process. In fact, many biological processes—notably homeostasis and switch mechanisms—are inherently nonlinear, with threshold effects for their perturbation to a degree sufficient to have health consequences. A rigorous examination of how additivity to background affects dose response for different modes of action should be undertaken before its general applicability is assumed.

The approach taken when extrapolating dose–response relationships to low doses has profound impact on risk-management decision making. If a change is proposed that is to be justified by invoking general principles, then the bearing of those principles needs to be rigorously articulated, well understood, and evaluated through broad discussion and debate. At present, this discussion has yet to occur.

## References

[b1-ehp-117-a141] Rhomberg LR, McDaniels TL, Small MJ (2004). Mechanistic considerations in the harmonization of dose-response methodology: the role of redundancy at different levels of biological organization. Risk Analysis and Society: An Interdisciplinary Characterization of the Field.

[b2-ehp-117-a141] White RH, Cote I, Zeise L, Fox M, Dominici F, Burke TA, White PD, Hattis DB, Samet JM (2009). State-of-the-science workshop report: issues and approaches in low dose–response extrapolation for environmental health risk assessment. Environ Health Perspect.

